# Children’s active school transportation: an international scoping review of psychosocial factors

**DOI:** 10.1186/s13643-023-02414-y

**Published:** 2024-01-30

**Authors:** Eva Savolainen, Anna-Karin Lindqvist, Katarina Mikaelsson, Lars Nyberg, Stina Rutberg

**Affiliations:** Department of Health, Education and Technology, Luleå University of Technology, 97187 Luleå, Sweden

**Keywords:** Active school commuting, Confidence in ability, Attitudes, Social support, Social norms

## Abstract

**Background:**

Over the last decades, the prevalence of AST has decreased significantly. Barriers to active school transport (AST) have been extensively examined in the literature, while psychosocial factors that facilitate AST have received less attention. To our best knowledge, there are currently no reviews on this subject. Therefore, the objective of this review was to scope the literature and identify published research about psychosocial factors related to AST.

**Methods:**

Systematic searches conducted in PubMed, Web of Science, TRID, Scopus, and ERIC resulted in a total of 1933 publications, and 77 of them were considered eligible for this review.

**Results:**

The results of the included articles were categorised into four psychosocial factors: confidence in ability, attitudes, social support, and social norms, which were all generally positively related to AST, with a few exceptions.

**Conclusion:**

The findings of this review indicate that these psychosocial factors may be important to consider when developing interventions and highlight that both children and parents should be involved in the process. This knowledge can serve as a valuable guide for developing interventions to promote AST. However, the evidence base supporting these psychosocial factors requires further investigation to fully understand how and when to incorporate them to maximise AST efficacy.

**Supplementary Information:**

The online version contains supplementary material available at 10.1186/s13643-023-02414-y.

## Introduction

Physical inactivity is a primary risk factor for non-communicable diseases [24]. Around 80% of children and youths do not engage in the recommended 60 min of daily physical activity [33]. Being physically active is a crucial determinant not only for physical health but also for mental, social, and environmental health [6]. Thus, the importance and urgency of health-promoting efforts to increase daily physical activity among children are evident and should be prioritised [118]. One suggestion for increasing daily physical activity is cycling or walking to school, also known as active school transportation (AST) [66]. However, the prevalence of AST has significantly decreased globally [6], which may be a result of, for example, changed distances and attitudes [90].

To address decreases in AST, a wide range of interventions have been launched, including walking school busses and safe routes to school [15, 41, 46, 72, 102]. Nevertheless, as shown by Larouche et al. [46] and Schonbach et al. [96], intervention trials have exhibited varied levels of effectiveness and quality. A thorough understanding of the factors associated with AST interventions should serve to increase their effectiveness and quality [46], among which psychosocial factors are highlighted as an important target area [47, 64]. In this review, psychosocial factors are defined as influences that affect a person psychologically or socially [110], and more specifically, we refer to intrapersonal and interpersonal characteristics towards AST, such as attitudes, self-efficacy, and norms [64].

There is a knowledge gap regarding the psychosocial factors affecting parents’ decisions concerning AST [101]. Also, according to Mertens and Ghekiere [64], the current evidence regarding the psychosocial factors related to children’s transport behaviour is limited. The growing body of literature on parent’s decision-making processes regarding AST provides some insight into this matter [2, 4]. A considerable amount of research has focused on barriers to AST [2, 4, 54]. While identifying barriers is imperative for developing effective interventions, it is insufficient for increasing AST [85, 101]. Towards that end, which factors facilitate AST should also be explored [54]. Parents are often the primary decision-makers regarding children’s transport modes. As such, their opinions are fundamental, however, children’s perspectives also greatly impact their use of AST [119]. Therefore, both perspectives must be considered to facilitate AST [80].

Previous reviews have focused on the physical and environmental attributes and social and sociodemographic characteristics associated with AST [39], but none have yielded a comprehensive understanding of the psychosocial factors specifically related to AST. Additionally, previous reviews have investigated the effectiveness of AST interventions [46, 72] but not the interventional effects on psychosocial factors related to AST. Furthermore, most of the reviews centred their analyses on quantitative studies,thus, there is a lack of knowledge about qualitative studies’ contributions to this matter. A comprehensive identification of the psychosocial factors reported by children and parents, therefore, represents valuable knowledge about their relation to AST. To the current knowledge base, this review adds a comprehensive review of the evidence-based information available on psychosocial factors related to AST. Therefore, the objective of this review was to scope the literature and identify published research about psychosocial factors related to AST.

## Methods

A common purpose of a scoping review is to explore the extent of a research area and identify key factors related to a concept [5, 68]. Therefore, this approach seemed best suited to address the aim of this review. In addition, a scoping review allows all study designs to be included [5]. The present review is presented in accordance with the 2018 PRISMA extension for scoping reviews checklist [109]. The protocol was established prior to this scoping review and is available upon request from the corresponding author.

### Eligibility criteria

Following Arksey and O’Malley [5], a set of eligibility criteria was defined using the population, concept, and context (PCC) framework [78]. The criteria were tested and discussed by the research team several times before the first screening and modified a few times thereafter due to increased familiarity with the literature. To be included in this review, papers needed to be peer-reviewed, examine and report on psychosocial factors that facilitate or promote AST, include children (6–18 years of age) and/or parents, and be written in English. We chose to only include research papers as AST due to the rapid increase of research in this relatively established field [90]. No date range limitation was applied. Papers were excluded if they focused on active transport in general or were published as conference material. The final version of the eligibility criteria is presented in Table 1.

**Table 1:** Overview of eligibility criteria

Inclusion/exclusion criteria		
***Population***	Inclusion	Children aged 6 to 18 years and their parents
	Exclusion	College or university students
***Concept***	Inclusion	Studies with AST as an outcome measure or central phenomenon of interest, where facilitating psychosocial factors among children and/or parents were investigated and reported with respect to how they affect or relate to AST. Studies addressing both facilitating/promoting and impeding psychosocial factors were also included
	Exclusion	Studies identifying AST as a means for other outcomes such as decreased obesity. Studies that only address impeding factors
***Context***	Inclusion	Active transportation to and from school
	Exclusion	General active travel or general mode choices
***Types of sources***	Inclusion	Peer-reviewed, empirical studies, any study design, written in English
	Exclusion	Study protocols, conference material, opinion papers, chapters, reviews, books

### Information sources and search

The choice of databases in this review were guided by the aim [5], striving to cover the interdisciplinary characteristics of AST research. We followed Arksey and O’Malley’s [5] recommendation to advise information specialists regarding the search process, including databases and search strategy. The informational specialist advised us not to use Google Scholar due the limitations regarding the Boolean operators and the personalised algorithm, a problem also identified by other researchers [34, 79], which makes it problematic to systematise and replicate a search strategy. We also investigated which search strategies that had been used in previous reviews of AST. The final search strategy was based on all these fore-mentioned considerations through a discussion within the research team, and the following databases were chosen to identify relevant articles: PubMed, Scopus, TRID, ERIC, and Web of Science.

The databases were searched in November 2020 and again in February 2022. An additional search was conducted to identify articles published between November 2020 and February 2022 due to the rapid and increasing pace of research in this area [90]. The PCC framework for scoping reviews [78] was the basis for the final search strategy, as presented in Table 2. The complete search strategy for an example database is presented in Table 3. All articles retrieved in the search were exported into EndNote, where duplicates were removed, and the remaining articles were exported to Rayyan for screening.

**Table 2:** Overview of search terms

***Population*** child* OR youth* OR adolescent* OR student* OR pupil*
**AND**
***Concept*** “active transport*” OR “active travel*” OR “active commut*” OR “active school transport*” OR “active school commut*” OR “active school travel*”
**AND**
***Context*** school*

^*^The asterisk was used as a wildcard to broaden the search terms

**Table 3:** Search strategy for the Web of Science

	**Search strategy**	**Results**
Date: 2020–11-03	**TOPIC:** (child* OR youth* OR adolescent* OR student* OR pupil*) *AND* **TOPIC:** (“active transport*” OR “active travel*” OR “active commut*” OR “active school transport*” OR “active school commut*” OR “active school travel*”) *AND* **TOPIC:** (school*) **Timespan:** All years. **Indexes:** SCI-EXPANDED, SSCI, A&HCI, CPCI-S, ESCI	1309
Limits:	None	

### Selection of sources of evidence

Two reviewers (E.S., K.M.) conducted an independent and blinded screening of titles and abstracts in the software Rayyan. The same reviewers blindly screened the full text of the articles to assess the relevance according to the eligibility criteria of the present review. The process proceeded with unblinding the screening and a calculation of agreement, which revealed a Cohen’s kappa value of 0.86, indicating perfect agreement according to Landis and Koch [45]. Disagreements and uncertainties regarding the selection of sources were settled by dialogue between the two reviewers (E.S., K.M.) or between all authors when required.

### Data charting process

Data from eligible studies were charted using a standardised data charting Excel form developed by two reviewers (E.S., K.M.) and approved by all authors. Two reviewers (E.S., K.M.) charted data from five articles to ensure that all relevant information was extracted. One of the reviewers (E.S.) independently charted the data, while the other (K.M.) verified the data’s accuracy.

### Data items

The reviewer (E.S.) extracted data on (1) article characteristics, including reference, country, aim, design/method, population, outcome variables, and theoretical framework; (2) psychosocial factors; and (3) main findings. The final version of the charting form can be found in Supplementary File 1. Inspired by Arksey and O’Malley [5], a thematic analysis of the extracted data was conducted, the results of which are presented in a narrative format.

## Results

**Fig. 1:**
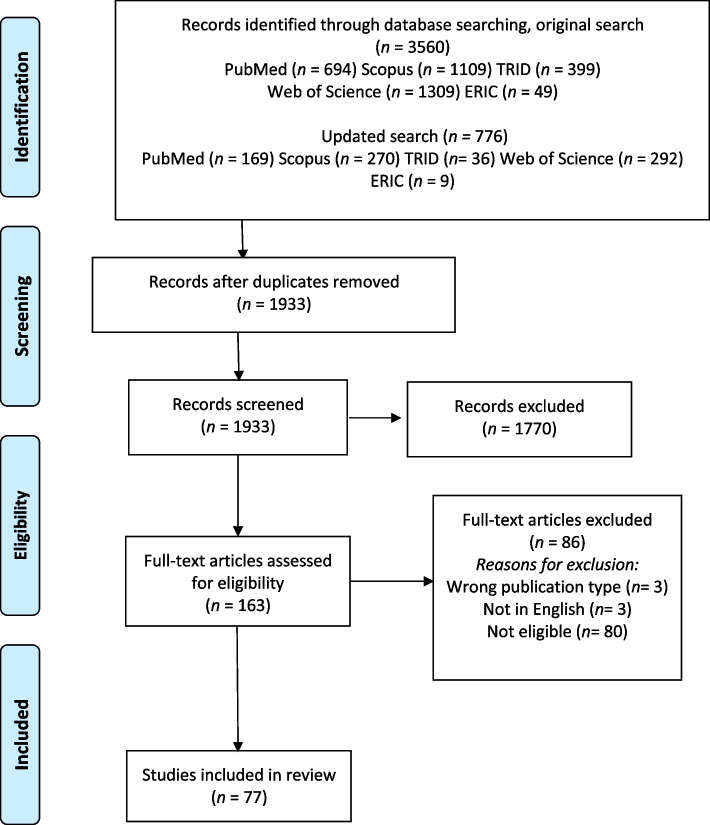
PRISMA flowchart of the review process

The main literature search resulted in 3560 publications, and the second search resulted in 776 publications; after the duplicates were removed, 1933 publications remained. After screening the titles and abstracts, 163 remained for full-text assessment of eligibility. During the full-text screening, 86 studies were excluded, and the remaining 77 articles were considered eligible for inclusion in this review. A flow chart of the screening process, as well as excluded full-texts and reasons for that, are provided in Fig. 1.

### Characteristics of sources of evidence

Among the 77 included articles, a cross-sectional (*n* = 49) or interventional study design (*n* = 12) were most used. Some studies included both children and parents (*n* = 23), others either children (*n* = 27) or parents (*n* = 23), and four also included teachers or school representatives. Some of the studies conducted with both children and parents also included other participants such as school staff or other adults. Studies were performed worldwide, with the majority in North America (*n* = 29), Europe (*n* = 29), Oceania (*n* = 15), two in South America, and one in Asia. One of the articles included two countries from different continents in their study. Publication year varied from 2006 to 2021 with most of the articles published between 2014 and 2021 (*n* = 64). Theoretical frameworks were included in 43 of the articles, whereas the social ecological model (*n* = 11), social cognitive theory (*n* = 9), and the theory of planned behaviour (*n* = 9) were the most used. An overview of the characteristics can be found in Table 4. The characteristics of each source of evidence in detail with references are presented in Supplementary File 2, and the results of each individual sources of evidence can be found in Supplementary File 3.

**Table 4:** Characteristics of sources

Characteristics of sources (*n* = 77)		Count (%)
**Study design**		
	Qualitative	10 (13%)
	Quantitative	63 (82%)
	Mixed method	4 (5%)
**Study participants**		
	Children and parents	23 (30%)
	Children	27 (35%)
	Parents	23 (30%)
	Children, parents, and other adults	4 (5%)
**Continent**		
	North America	29 (38%)
	Europe	29 (38%)
	Oceania	15 (19%)
	South America	2 (3%)
	Asia	1 (1%)
	Multiple continents	1 (1%)
**Publication year**		
	2006–2013	13 (17%)
	2014–2021	64 (83%)
**Theoretical framework**^a^		
	Social ecological model	11 (14%)
	Social cognitive theory	9 (13%)
	Theory of planned behaviour	9 (12%)
	Self-determination theory	3 (4%)
	Multiple frameworks	4 (7%)
	Theory of reasoned action	1 (1%)
	Ecological and cognitive active commuting (ECAC) model	1 (1%)
	Pathway model	1 (1%)
	Social marketing	1 (1%)
	Social norms approach (SNA)	1 (1%)
	The travel socialisation framework	1 (1%)
	Integrative model of behaviour prediction	1 (1%)

^a^The percentage in the theoretical framework section is calculated on the total number of articles (*n* = 77)

### Synthesis of the results

In the present review, we aimed to scope the literature and identify studies about psychosocial factors related to active school transportation. The results indicated that psychosocial factors relating to AST have been explored in various ways, indicating that confidence in ability, attitudes, social support, and social norms could be important factors for AST. The result also points toward some inconsistencies within this area and that demographic and modes of active travel can mediate the significance of the psychosocial factors presented in this review.

The synthesis of the result is further divided into two sections. The first is based on psychosocial factors reported by children related to AST (Table 5 and 6). The second section is based on psychosocial factors reported by parents related to their children’s use of AST (Table 7 and 8). Both sections describe whether and how these psychosocial factors are affected by interventions (Table 9 and 10).

**Table 5:** Quantitative studies on psychosocial factors among children relating to AST

Psychosocial factor	Positively related to AST (*n*)	No relation to AST (*n*)	Negatively related to AST (*n*)
Confidence in ability	***n* = 8** [10, 55, 69, 99, 103, 108, 117, 122]	***n* = 5** [13, 61, 62, 71, 111]	***n* = 0**
Attitudes	***n* = 14** [11, 22, 27, 38, 42, 50, 58, 59, 69, 86, 103, 105, 117]	***n* = 6** [9, 13, 71, 99, 111, 122]	***n* = 0**
Social support	***n* = 11** [10, 12, 14, 22, 28, 50, 53, 71, 75, 99, 117]	***n* = 5** [13, 17, 31, 84, 111]	***n* = 0**
Social norms	***n* = 9** [13, 17, 36, 42, 53, 69, 103, 111, 116]	***n* = 1** [122]	***n* = 0**

**Table 6:** Qualitative studies on psychosocial factors among children relating to AST

Psychosocial factor	Described relation to AST
Confidence in ability	No data retrieved
Attitudes	• Parental attitudes towards active travel varied and could have either a positive or negative influence over their child’s behaviour [44] • Perceived benefits such as feelings of calmness, being in a good mood, time to think, having fun, socialising, feeling healthy and independent as well as helping the environment were related to active travel to school [25, 37, 44, 91, 93]
Social support	Social support positively related to AST: • Travelling with friends or parents [44, 83] • Parental encouragement [83] • Teachers’ encouragement [94] • A sense of togetherness [51]
Social norms	Social norms related to AST: • Parents choosing active transport facilitated AST [83] • Peers using AST positively related to AST [93] • Social norms negatively correlated with cycling to school since cycling was considered to be for sporty people due to the hilly landscape for some of the participants in the study by Hopkins and Mandic [37]

**Table 7:** Quantitative studies on psychosocial factors among parents relating to their children’s use of AST

Variable	Positively related to AST (*n*)	No relation to AST (*n*)	Negatively related to AST (*n*)
Confidence in ability	***n* = 11** [26, 29, 55, 56, 61, 62, 73, 97, 98, 100, 107]	***n* = 2** [40, 114]	***n* = 0**
Attitudes	***n* = 15** [18, 29, 48, 56, 63, 74, 75, 87, 89, 97, 100, 120, 121, 123, 124]	***n* = 6** [26, 40, 59, 73, 98, 114]	***n* = 0**
Social support	***n* = 16** [13, 23, 26, 35, 57, 60, 63, 75, 76, 82, 87, 88, 100, 112, 123, 124]	***n* = 1** [121]	***n* = 0**
Social norms	***n* = 9** [29, 40, 73, 74, 88, 89, 97, 98, 100]	***n* = 1** [114]	***n* = 0**

**Table 8:** Qualitative studies on psychosocial factors among parents relating to their children’s use of AST

Variable	Described relation to AST
Confidence in ability	No data retrieved
Attitudes	Perceived benefits described as reasons for involving their children in AST: • Regular exercise, spending time together [1, 25, 70] • Better health [25, 70, 81]
Social support	Social support facilitating AST: • A community feeling [81] • If their child was accompanied [92]
Social norms	Social norms related to parents’ decision-making regarding AST: • What was viewed as normal and natural behaviour [81] • Observing how other parents act as well as seeing other children manage AST [30]

**Table 9:** Interventional effects on psychosocial factors and AST among children

		**Reported effects**					
**Reference**	**Intervention**	**Confidence in ability**	**Attitudes**	**Social support**	**Social norms**	**Children’s AST behaviour**	**Comment**
[16]	Baseline and follow-up data on a multicomponent intervention involving improvements of non-curricular PA through changes in the physical and organisational environment supported by educational activities	NA	No effect	No effect	NA	Both the intervention and comparison group increased their AST, and no significant differences were reported	
[36]	Baseline and follow-up data on a safe route to school intervention (SRTS) involving non-infrastructure (encouragement) and infrastructure (engineering) intervention	Positive effects on self-efficacy in the short term	NA	Positive effect on parental support	No effect	Positive effects on the short but not long term	Non-infrastructure funding appears to have slightly negative effects on AST over time compared with matched schools without funding
[103]	Baseline and follow-up data on an intervention involving three elements: (i) information, (ii) reflection, and (iii) action	Positive effect of PBC in the test group	Positive effect on attitudes in the test group	NA	No effect	Positive effect on intentions in the test group	Difference models show that changes in attitude, subjective norm, and PBC accounted for 29% (car passenger) to 92% (walking) of the variance in changes in intention

**Table 10:** Interventional effects on psychosocial factors among parents and their child’s AST

		**Reported effects**					
**Reference**	**Intervention**	**Confidence in ability**	**Attitudes**	**Social support**	**Social norms**	**Children’s AST behaviour**	**Comment**
[36]	Baseline and follow-up data on a safe route to school (SRTS) intervention, involving non-infrastructure (encouragement) and infrastructure (engineering) intervention	Positive effect on parents’ self-efficacy among parents from infrastructure schools	NA	Positive effects on parental support	NA	Positive effects in the short but not on long term	Non-infrastructure funding appears to have slightly negative effects on AST over time compared with matched schools without funding
[51]	Free-form questionnaire and photovoice data on a gamification-based intervention involving curriculum assignments during AST	NA	Positively affected parents’ attitudes	NA	NA	The results show that the intervention motivated the students to use AST	
[91]	Focus-group data on a gamification-based intervention involving curriculum assignments during AST	NA	Positively affected parents’ attitudes	NA	NA	The children became highly motivated and put additional effort into AST	
[92]	Mixed method data on a gamification-based intervention involving curriculum assignments during AST	NA	Positively affected parents’ attitudes	NA	NA	NA	

### Psychosocial factors among children relating to AST

#### Confidence in ability

Confidence in ability was mainly described by two constructs: self-efficacy and, in some cases, perceived behavioural control (PBC). Both refer to confidence in ability to perform AST, and PBC also refers to the degree of control/autonomy and the expectation on how easy it is to perform AST. Most of the articles reported a positive relationship between children’s confidence in ability and AST, while several found no relationship (Table 5). However, among the articles reporting a positive relationship, Trapp et al. [108] found that this was only among girls. Furthermore, Sims and Bopp [100] found a positive relationship between PBC and AST but not between parental self-efficacy and AST. Emotional states and modelling positively associated with children’s self-efficacy [55].

Table 9 shows that interventions like changes in built environment or encouraging AST, including information, reflection and action, and bicycle training were reported to be positively related to children’s self-efficacy [36] and PBC [103]. However, the intervention that measured AST showed no interventional effects on AST [16].

#### Children’s attitudes

Attitudes were described as an overall positive or negative evaluation of performing AST and could include both an affective (enjoyable) and an instrumental (beneficial) component. As shown in Table 5, most studies reported a positive relationship between attitudes and AST, while several did not found this relationship. Among the articles reporting a positive relationship, Leslie et al. [50] found a positive relationship between enjoyment and AST among boys only. Also, Rodriguez et al. [86] reported a positive relationship between attitudes and AST but found no relationship between perceiving AST as fun and AST. Stark, Meschik et al. [104] further reported a positive relationship between attitudes and walking, but no relationship between attitudes and cycling were found. Moreover, attitudes towards AST were reported to be positively associated with social interactions [44].

One intervention study, including information, reflection, and action, had a positive effect on attitudes and reported positive changes in intentions to use AST as well. On the other hand, in an interventional study, improvements of non-curriculum PA had no effect neither on attitudes nor children’s AST (Table 9). Furthermore, perceiving AST as fun was described as important for the willingness to use AST in qualitative intervention evaluations [38, 94].

#### Children’s perceived social support

Social support was described as comprising a sense of togetherness, encouragement, practice, appraisal, and invitations to perform AST. Social support from peers, parents, and teachers were positively related to children’s AST in most of the studies; however, several studies did not observe any evidence of such a correlation (Table 5). Among the articles reporting a positive relationship, Leslie et al. [50] only found a positive relationship among boys, and they further concluded that high degrees of encouragement were negatively associated to AST from school among girls. Also, Camargo [12] only found a positive relationship between parental support and AST in boys, while peer support was positively related to AST in both boys and girls. Long et al. [53] only found a positive relation between parental support and AST but not between peer support and AST, while Nunes de Oliveira et al. [71] observed the opposite.

From qualitative studies (Table 6), a sense of togetherness, as well as encouragement from friends, classmates, teachers, and parents, positively related to children’s AST [38, 44, 51, 83, 91, 94].

One intervention study, including changes in the built environment, had a positive effect on social support, while another intervention study, including improvements in non-curricular PA, showed no effects on social support (Table 9). However, neither of these interventions influenced AST (Table 9).

#### Children’s perceived social norms

Social norms were described as the perceived prevalence of friends and parents’ cycling or walking (modelling), perceived approval of AST, believing that others want them to perform AST and expectations on how others will evaluate it. Social norms and modelling positively related to children’s AST in most of the included articles, while one found no associations (Table 5). Among the articles reporting a positive relation, Van Dyck et al. [111] reported a positive relationship between modelling and AST but found no relationship between social norms and AST.

What other people think and do in terms of AST was described in the qualitative studies to influence the children as well [37, 93].

None of the intervention studies reported any effects on social norms (Table 9).

### Psychosocial factors among parents related to their children’s AST

#### Parents’ confidence in their child´s ability

Confidence in a child’s ability was described by two constructs: self-efficacy and PBC. Self-efficacy refers to parents’ confidence in their children’s ability to perform AST. PBC refers to parents’ beliefs regarding their own and their children’s ability and personal control over AST, as well as their perceptions of the ease or difficulty of performing AST based on extrinsic factors. Parents’ perceived confidence in their child’s AST positively related to their child´s AST in most studies, while a few reported no relationship (Table 7). Among the studies reporting a positive relation, Forsberg et al. [29] reported that when PBC was divided into impeding and facilitating factors, only the latter was important for parents. Sims and Bopp [100] further reported that parents’ PBC was positively related to AST but found no relationship between parents’ self-efficacy and AST. Moreover, Lu et al. [55] reported that parents’ perceived confidence in their child’s ability had a stronger influence on AST than the child’s own self-efficacy and that children’s self-efficacy and parents’ perceived confidence in their child’s ability correlated.

One qualitative intervention study based on gamification elements and one quantitative intervention study that involved changes in the built environment or encouragement for AST were reported to be positively related to parent’s confidence in their child’s AST and children’s use of AST as well (Table 10). Another intervention study that included bicycle training concluded a positive effect on parental confidence in ability but did not investigate effects on AST (Table 10).

#### Parents attitudes

Attitudes were described as parents’ reflection of AST as favourable or unfavourable and as a positive or negative evaluation of performing AST. Attitude is comprised of both instrumental (i.e. believing that AST is good for you) and affective (i.e. enjoying AST) components. Most of the studies reported a positive relationship between a parent’s attitude and their child’s use of AST, while a few found no relationship (Table 7 and 8). Among the studies reporting a positive relation, Forsberg et al. [29] concluded that positive attitudes were an important factor for parents’ intention to let their children cycle to school, but not regarding parents’ intention to let their children walk to school. Also, Corral-Abos [18] only found a positive relationship between children’s AST and mother’s attitudes, but not fathers’. Another study could not support a significant positive relationship between affective attitudes and intention to use AST, however, the study unexpectedly found that instrumental attitudes had a significant negative effect on the intention to use AST [98]. Furthermore, peer support was positively related to parental attitudes [121], and social norms were positively related to parents’ attitudes [40].

Three qualitative interventional studies concluded that parents’ attitudes were positively affected by a gamification-based intervention, which may have also positively influenced the children’s AST [51, 91, 92].

#### Parents perceived social support

Social support was described in the articles as verbal encouragement, emotional support, facilitation, and modelling, which, in this section, refers to parents’ perspectives on social support regarding both their support and peer support. All of the studies found that parents perceived social support positively correlated to children’s AST (Table 7). However, among those that reported a positive association, Van Kann et al. [112] only found a positive relationship between being a parent who actively travels and AST, but not between PA support and AST.

The qualitative study by Porskamp et al. [81] describes how a community feeling could make parents more likely to let their child use AST.

No effects on social support due to interventions were reported (Table 10).

#### Parents perceived social norms

Social norms were described both as parents’ perception of whether people of importance accept or reject AST and as a perceived social pressure to perform or not perform AST. Two different types of social norms were also distinguished among the articles: descriptive (i.e. an individual’s perception of AST in the majority) and injunctive (i.e. if AST feels right based on morals or beliefs). Parental perceived social norms were positively related in most of the studies, while one found no relationship (Table 7). Among the studies reporting a positive association, Jing et al. [40] only found a positive relationship between descriptive norms and AST, while no significant relationships to injunctive norms were found.

From the qualitative study by Forsberg et al. [30], the perception of being a good parent by driving the child safely to school is important, while seeing other children use AST makes an active choice more likely. Similarly, in their qualitative study, Porskamp et al. [81] describe how shared norms affected parents’ perception regarding which mode of transportation is considered normal.

No effects on social norms due to interventions were reported (Table 10).

## Discussion

The objective of this review was to scope the literature and identify published research about psychosocial factors related to AST. In doing so, we collected valuable knowledge about factors of importance when developing interventions to promote AST. The number of studies we found indicated that this issue has received quite a lot of research interest. The results of the included articles were categorised into four psychosocial factors: confidence in ability, attitudes, social support, and social norms, all of which were generally positively related to AST, with a few exceptions. Since most studies were cross-sectional, no causal relationships could be established based thereon. However, some intervention studies indicated that most of the psychosocial factors could be affected by interventions, and a few of them also reported an interventional effect on AST.

The major contribution of this review to the existing literature is the findings of psychosocial factors positively related to AST, which are similar to findings regarding PA [19, 20, 32, 43, 52, 67, 77, 106]. These results align with previous research regarding barriers to AST, such as lack of social support and confidence in ability [4]. Social support is also linked to a higher likelihood of perceiving it as safe, which is an important aspect among parents when it comes to letting their children use AST [4]. Crawford et al. [21] further emphasise that children are being influenced by their parent’s concerns and stress the importance of providing opportunities for children to practice skills for safe travel.

The result showed that the psychosocial factors are positively related to AST both from the child's and parents’ perspectives; thus, involving both of these stakeholders is crucial when developing interventions to promote AST [66, 113]. Our results show that parents’ choice of their child’s travel mode is partly explained by their attitude towards AST, their confidence in their child’s ability and social norms, and social support, which can support its use. In addition, the results also revealed that parents’ attitudes towards AST could be mediated by perceived peer support and social norms. This assimilates with the theory of planned behaviour, which suggests that feedback effects on attitudes can be derived from social norms and can positively or negatively influence attitudes [3]. Thus, if parents get positive reactions from others regarding letting their children use AST, it could positively influence their attitudes. Another example is that children’s confidence in their ability can be mediated by modelling, which ties in well with social cognitive theory because modelling, in turn, refers to a vicarious experience that can contribute to an individual’s self-efficacy [7]. Thus, theoretical underpinnings that recur in the literature are likely valuable in future research regarding AST [54, 65, 101].

Most of the studies did not separate walking and cycling into two different behaviours. However, those that did report differences between cycling and walking regarding their relation to psychosocial factors. Differences, such as cultures, demographics, distance, varied landscapes, and traffic, are likely to be context-related. Thus, separating cycling and walking as two different behaviours is valuable in future research to understand possible differences between the behaviours and how to promote them [54]. The findings also differed by sex in several studies, e.g. regarding social support from parents, which proved worthy of additional consideration as previous research has reported a higher perceived vulnerability concerning stranger danger for girls than boys [49].

Even though there were a limited number of studies exploring interventional effects on psychosocial factors, the result indicated that most of the parental and child psychosocial factors could be positively affected by an intervention. Unfortunately, the interventional studies that also included measures of effects on AST are rare, and those that do describe varied results, such as no effect or short-term effects. Previous reviews have highlighted the importance of more research on effective interventions within the AST research field to achieve long-term effects [46, 115]. In line with Larouche et al. [46], we would like to stress the need to investigate mediators of AST. This review provides knowledge about psychosocial factors that may offer guidance for further investigations regarding mediators of AST, which could be important to target with interventions.

### Limitations and strengths

This review presents an overview of current knowledge in accordance with the research aim; however, considering the reasons for a scoping review, the results have less depth but contribute to a comprehensive picture of the issue at hand. Informed by the methodological framework for scoping reviews, we applied a systematic, rigorous, and transparent methodology to capture a broad range of the psychosocial factors positively related to AST. The limitations of this research include the use of articles published in English only, and typically for scoping reviews, the quality of included articles was not assessed [68, 5]. Also, although the psychosocial factors presented in this review were positively related to AST in most cases, interpretations of one factor leading to an increase in AST should be avoided due to the cross-sectional design of most of the studies.

Following our search strategy and making the choice not to include unpublished literature might have an impact on publication bias [34, 95]. Google Scholar is considered to be a valuable source of non-commercially published literature such as academic theses and governmental reports [34], and combining databases such as Web of Science, Medline, and Embase with a search engine like Google Scholar might be the optimal choice for systematic reviews [8]. It is possible that we in this scoping review have missed some articles since non-commercial literature, such as academic theses and conference material, were not included [34]. However, we conducted two searches on different time points, and the unpublished literature in the first round is likely to have been published by the second round. Moreover, the main points of this review did not change with the extra articles found in the second round.

Nevertheless, the psychosocial factors presented in this review are based on studies with diverse research designs conducted in five continents representing various prerequisites for AST, including cultural contexts, policies, and climate aspects. Another strength of this study is that we started with broad search terms in several databases, which minimises source bias [95]. Moreover, the detailed information on inclusion and exclusion criteria provided in this paper and the in-depth description of the search terms reduce selection and scope bias and enhance transparency [95].

## Conclusion

This review provides an overview of the existing research about psychosocial factors related to AST, and the results showed that confidence in ability, attitudes, social support, and social norms are psychosocial factors that are positively related to AST and, therefore, likely represent factors critical to successful AST interventions. The findings also show the importance of involving both children and parents in promoting AST and include several examples of interventions that positively affected psychosocial factors. This knowledge could serve as a valuable guide in developing effective interventions to promote AST. However, the evidence base of these psychosocial factors needs to be further investigated to fully understand how and when they should be utilised in interventions.

### Supplementary Information

**Additional file 1.** Final version of data chartingR4.

**Additional file 2.** Characteristics of each sourceR4.

**Additional file 3.** Results of each source of evidenceR4.

## Data Availability

All data generated or analysed in this study are included in this manuscript and its supplementary files.
